# Evaluation of human first trimester decidual and telomerase-transformed endometrial stromal cells as model systems of in vitro decidualization

**DOI:** 10.1186/1477-7827-9-155

**Published:** 2011-12-07

**Authors:** Leila Saleh, Gerlinde R Otti, Christian Fiala, Jürgen Pollheimer, Martin Knöfler

**Affiliations:** 1 Department of Obstetrics and Fetal-Maternal Medicine, Reproductive Biology Unit, Medical University of Vienna, A-1090 Vienna, Austria; 2 Gynmed Clinic, A-1150 Vienna, Austria

## Abstract

**Background:**

Decidualization, the differentiation process of maternal uterine stromal cells into secretory decidual cells, is a prerequisite for successful implantation and progression of pregnancy. For in vitro differentiation mostly primary human endometrial stromal cells (HESC) isolated from uterine samples after hysterectomy for benign gynaecological diseases are utilised. However, a continuous supply of endometrial tissue is often lacking. Hence, we analysed whether cultivated human decidual stromal cells (HDSC) prepared from first trimester pregnancy terminations may represent an alternative model system for in vitro decidualization. Moreover, based on the expression of critical marker genes these cells were compared to a previously established endometrial stromal cell line during in vitro differentiation.

**Methods:**

HDSC isolated from decidual tissue attached to first trimester placentae, and telomerase-transformed human endometrial stromal cells (THESC) were characterised by immunofluorescence and differentiated in vitro using either cyclic adenosine monophosphate (cAMP) and/or estrogen (E2)/progesterone (P4). Proliferation was measured by analyzing cumulative cell numbers. Expression of mRNAs encoding progesterone receptor (PR), prolactin (PRL), insulin-like growth factor binding protein-1 (IGFBP1), and Dickkopf-1 (DKK1) was evaluated using quantitative PCR after 3, 6, 9 and 12 days of in vitro differentiation. PRL and IGFBP-1 protein expression was investigated by enzyme-linked immunosorbent assay (ELISA) and Western blotting, respectively. Furthermore, forkhead box O1A (FOXO1A), a critical transcription factor in decidualization, was analysed by immunofluorescence and Western blotting at two different time points of differentiation.

**Results:**

Treatment with cAMP provoked morphological changes and growth arrest of THESC and HDSC, the latter showing loss of cells after 6 days of treatment. E2P4 stimulation did neither affect cell morphology nor proliferation of THESC and HDSC. Upon cAMP stimulation PR mRNA was suppressed in HDSC but not in THESC, whereas E2P4 did not alter transcript levels in both cell types. Protein expression of PR-A and PR-B was detectable in HDSC and diminished under cAMP, whereas THESC failed to produce the nuclear receptors. Supplementation of cAMP induced mRNA and protein expression of PRL and IGFBP-1 in both cell types at day 3, 6, 9, and 12 of treatment. In HDSC stimulation with E2P4 increased PRL and IGFBP-1 mRNA and protein production, whereas hormone treatment did not induce the two factors in THESC. E2P4 increased DKK1 mRNA at all time points in HDSC and cAMP provoked induction at day 9 and 12 of differentiation. In contrast, cAMP suppressed DKK1 mRNA in THESC, whereas E2P4 was ineffective. In both cell types combined treatments with cAMP and E2P4 provoked higher expression levels of PRL and IGFBP1 mRNA and protein as compared to cAMP stimulation alone. FOXO1A protein and its nuclear abundance were increased by cAMP in both cell types. However, reduction of its nuclear localisation upon E2P4 treatment could only be observed in HDSC.

**Conclusion:**

Both HDSC and THESC may represent suitable model systems for cAMP-dependent in vitro decidualization. Since cAMP decreases cell viability of HDSC after 6 days of incubation, this substance should be preferentially used in short-term experiments. Progesterone treatment of THESC might not be applicable since these cells lack progesterone response and PR protein. In contrast, stimulation of PR-expressing HDSC with E2P4 or cAMP/E2P4 may represent an appropriate protocol for human in vitro decidualization inducing and maintaining expression of critical marker genes in a time-dependent manner.

## Background

Differentiation of estrogen-primed, uterine stromal cells into stromal cells of pregnancy, termed decidualization, starts in the secretory phase of the menstrual cycle due to rising progesterone levels. In humans the process occurs independently of an implanting blastocyst but is only maintained upon pregnancy, since absence of a conceptus provokes shedding of the decidualized endometrial layer and menstruation. Decidualization involves profound changes in cellular function, morphology and gene expression. Typically, spindle-shaped uterine stromal cells transform into polygonal, epithelial-like decidual cells producing characteristic hormones, growth factors and cytokines such as PRL [1], tissue factor [2], IGFBP1 [3], interleukin (IL)-15 [4] or IL-11 [5]. Secreted factors of the decidua are thought to fulfil numerous functions in pregnancy such as regulation of placental trophoblast implantation and invasion, recruitment and differentiation of uterine natural killer (NK) cells and protection against immune-mediated damage or oxidative stress [6-8]. Moreover, decidual stromal cells actively participate in remodelling of the extracellular matrix of the uterus by expressing diverse extracellular matrix (ECM)-proteins such as collagen IV, fibronectin, laminin and alpha smooth-muscle actin, the latter provoking myofibroblast-like properties of the cells [9,10]. The crucial role of decidual cells in pregnancy is further emphasized by the fact that gene knock-out of different decidual proteins in mice caused failures in implantation, infertility or intrauterine lethality [11].

Although the molecular mechanisms of decidualization are still poorly understood, various critical factors controlling differentiation and decidua-specific hormone expression have been unravelled. Transcription factors such as HoxA-10, HoxA-11, FOXO1A, Stat5 and C/EBPβ were shown to control promoter activity of decidual PRL and/or IGFBP1 in a differentiation-dependent manner [12]. Moreover, steroid hormones and their nuclear receptors, estrogen receptor (ER) and PR, play a pivotal role in decidual differentiation, since lack of these genes impairs implantation and decidualization [8]. Accordingly, progesterone is thought to be necessary to maintain the decidualized phenotype of the estrogen-primed endometrium. However, progesterone alone is a weak inducer of decidualization in primary HESC and the combined action of progesterone and cAMP is required for maximal and sustained expression of decidual marker genes [12]. For example, the decidual prolactin promoter lacking a palindromic PR recognition sequence, can only be stimulated with progesterone when cultures were pre-treated with exogenous cAMP suggesting that activation of the proteinkinase A pathway may enhance the transcriptional activity of PR through modification of PR and its co-activators [13]. Critical ligands such as relaxin, prostaglandin E2 or gonadotrophins are thought to activate cAMP-dependent signalling in these cells [14]. On the other hand, progesterone treatment provokes elevation of endogenous cAMP levels in decidualizing stromal cells [15] suggesting complex cross-talk between the two signalling pathways.

Given the fact that cAMP and steroid hormones are critically involved in uterine stromal cell differentiation, numerous protocols using exogenous cAMP analogues and/or estrogen/progestational hormones, such as P4 or medroxyprogesterone acetate (MPA) in vitro have been developed [14]. As cellular model for human in vitro decidualization HESC, which have been isolated from uterine samples and biopsies after hysterectomy for benign gynaecological conditions, such as myomas, or patients undergoing tubal ligation, are widely utilised [16,17]. However, availability of physiological, endometrial tissue might be limited provoking the need for alternative sources and model systems. Indeed, different cell lines have been developed which can be utilised for cAMP-dependent differentiation but often lack P4 responsiveness [18,19]. Hence, we herein analysed stromal cells which have been isolated from decidual tissue of first trimester pregnancy terminations as a possible model system of cAMP and/or E2P4-induced decidualization. Moreover, a commonly used, telomerase-transformed cell line, THESC [20], was investigated in parallel with respect to cAMP/steroid hormone-dependent differentiation and expression of decidual marker genes.

## Methods

### Isolation and cultivation of primary human decidual stromal cells

Decidual specimen were obtained from legal abortions of healthy first trimester pregnancies (between 8^th ^and 11^th ^week of gestation, n = 13) with the permission of the ethical committee of the Medical University of Vienna. After separation of decidual tissues from the placenta, samples were washed in phosphate buffered saline to remove blood clots and subsequently minced in 3 mm^3 ^pieces in a small volume of Hank's balanced salt solution (HBSS, Sigma-Aldrich, St. Louis, MO). Tissue fragments were collected in a T-75 cell culture flask and covered with 50 ml digestion solution. Primary human decidual stromal cells were prepared by enzymatic dispersion using 2 mg/ml collagenase I (484 IU/ml, Invitrogen, Paisly, UK) and 0.5 mg/ml DNAse I (Sigma-Aldrich) in HBSS containing 25 mM HEPES. After digestion (60 minutes at 37°C) in a shaking water bath, supernatant containing dispersed cells was filtered through a 70 μm nylon mesh (BD Biosciences, Bedford, UK) to remove larger tissue fragments. Residual undigested tissue was further incubated for 60 minutes in 25 ml of digestion solution. Subsequently, supernatants were pooled, centrifuged at 1000 rpm for 5 minutes and pellets were washed twice in HBSS. Isolated cells were seeded in phenolred-free DMEM/F12 medium (Invitrogen) supplemented with 10% heat-inactivated fetal bovine serum (PAA Laboratories GmbH, PaschingAustria), 1% ITS+ Premix (BD Biosciences), and 50 μg/ml Gentamicin (Invitrogen) and cultivated at 37°C, 5% CO_2 _and 95% humidity. Purity of HDSC was routinely examined by immunofluorescence using antibodies against vimentin and cytokeratin 7 detecting stromal, epithelial and endothelial cells, respectively (see below). Cultures were negative for CD31 (1 μg/ml; Abcam, Cambridge, UK), HUVEC were used as a positive control.

### Re-decidualization of primary human decidual stromal cells

For in vitro decidualization isolated HDSC were utilized between passage three and five. Briefly, cells were confluently seeded in 24-well plates (2.5 × 10^5^/cm^2^) and stimulated on the next day with 0.5 mM 8-Bromo-cAMP (Sigma-Aldrich) and/or 10 nM estrogen (E2; 17β-estradiol-acetate; Sigma-Aldrich)/1 μM progesterone (P4; 4-pregnene-3,20-dione; Sigma-Aldrich) for 3, 6, 9 and 12 days. HDSC kept under the same culture conditions but without addition of cAMP or E2P4 served as non-stimulated controls. Every third day medium was changed and stimuli were refreshed. Supernatants were collected, immediately frozen in liquid nitrogen and stored at -80°C for further analyses. Morphological changes were daily monitored with a phase contrast microscope and photographs were taken using a digital camera (C-4040 Zoom, Olympus, Vienna, Austria) for documentation. In addition, total protein and RNA were extracted from decidualizing cultures or non-stimulated controls at indicated time points.

### Cultivation and decidualization of THESC

The telomerase-immortalised endometrial stromal cell line THESC [20] was purchased from American Type Culture Collection (CRL-4003; Wesel, Germany) and cultivated as described by the supplier. Briefly, cells were cultured in phenol red-free DMEM/F12 medium (Invitrogen) supplemented with 10% heat-inactivated fetal bovine serum (PAA Laboratories GmbH), 1% ITS+ Premix (BD Biosciences) and puromycin (5 μg/μl; Invitrogen). In vitro decidualization of THESC was performed as described above for HDSC.

### Immunofluorescence of decidual tissue and cultured cells

First trimester decidual tissues (n = 3 between 8^th ^and 10^th ^week) were washed with phosphate buffered saline (PBS) and fixed for at least 4 hours in 4% paraformaldehyde. After embedding serial sections (3 μm) were prepared on a Microm HM 355S microtome and mounted on silanized microscope slides. Subsequently, slides were dried for 15 minutes at 55°C and dewaxed in xylol for 10 minutes. Finally sections were slowly rehydrated in decreasing concentrations of ethanol (100%, 96% and 70%) and gathered in deionised water. Antigen retrieval was performed using 0.05% citraconic anhydride solution pH 7.0 (Thermo Fisher Scientific, Waltham, MA) at 98°C for 20 minutes. Slides were allowed to cool down for 30 minutes at room temperature before they were washed three times with PBS and clamped with plastic cover slips (Shandon, Frankfurt, Germany). Unspecific binding sites were blocked with 100 μl 0.05% fish skin gelatin from cold water fish (Sigma-Aldrich)/PBS for 30 minutes at room temperature and 100 μl primary antibodies diluted in blocking solution were administered. After overnight incubation at 4°C slides were washed (100 mM TrisCl pH 7.5, 50 mM NaCl, 0.05% Tween20) and covered with 100 μl secondary antibodies diluted in blocking buffer. After one hour sections were washed again and counterstained with 100 μl DAPI (Roche Diagnostics GmbH, Vienna, Austria) diluted 1:1000 in PBS for 10 minutes. After a final washing step, slides were removed from the staining chamber and mounted with Fluoromount-G (Southern Biotechnology, Birmingham, UK). Sections were dried overnight and subsequently analysed by fluorescence microscopy using a BX 50 Microscope (Olympus). For immunofluorescence of HDSC and THESC, cells were seeded on 8-well chamber slides, fixed with 4% paraformaldehyde for 10 minutes, washed, and permeabilized with 0.1% Triton-X 100 (Sigma-Aldrich) for 5 minutes. Blocking, incubation with primary and secondary antibodies and counterstaining with DAPI were done as described above. The following primary and secondary antibodies were used: mouse anti-human cytokeratin 7, OV-TL12/30, Dako, 1:100; rabbit anti-human vimentin, H-84, Santa Cruz Biotech. 1:100; rabbit anti-human FOXO1A, C29H4, Cell Signaling Technology, Danvers, MA, 1:100; goat-anti-mouse Alexa Fluor 488, goat-anti-rabbit Alexa Fluor 568 or goat-anti-rabbit Alexa Fluor 488. All secondary antibodies were diluted 1:500. Analysis of signals was carried out by fluorescence microscopy (Olympus BX50) and digitally photographed. To determine FOXO1A protein expression digital images (four different fields for each condition) were counted using Cell^P Soft Imaging System (Olympus). Subsequently the ratio of FOXO1A-positive nuclei to DAPI-stained nuclei was calculated.

### Proliferation assay

To assess proliferation during decidualization cumulative cell numbers were measured. Primary HDSC and THESC were confluently seeded in duplicates (2.5 × 10^5^/cm^2^; 12-well plates), respectively. On the next day cultures were stimulated with cAMP or E2P4. Subsequently, cultures were trypsinized after 3, 6, 9 and 12 additional days and cell numbers were counted using a CASY Cell Counter (Schärfer System, Reutlingen, Germany).

### RNA extraction and quantitative real-time PCR

Total RNA was extracted by direct lysis in the culture dishes using TriFast Reagent (PeqLab, Erlangen, D) according to the manufacturer's instructions. RNA amount and integrity were evaluated using the Agilent Bioanalyzer 2100 (Agilent, Palo Alto, CA). 2 μg RNA were reverse transcribed using 200U of RevertAid H Minus M.MuLV Reverse Transcriptase (Fermentas, St.Leon-Rot, D), 0.2 μg Hexanucleotide Mix (Roche Diagnostics GmbH, Vienna, Austria), 0.5 μl RNaseOUT (Recombinant Ribonuclease Inhibitor, Invitrogen) and 0.5 mM dNTP (Promega, Madison, WI) in final volume of 20 μl. Quantitative real-time PCR was performed on the ABI 5700 Sequence Detection System (Applied Biosystems, Carlsbad, CA) using Taq Man Gene Expression Assays (TaqMan Universal PCR Master Mix, 20× Taq Man Gene Expression Assay Mix form PRL (Hs 00168730_m1), IGFBP1 (Hs 00426285_m1), DKK1 (Hs 0018740_m1), PR (Hs01556707_m1) and TATA-box binding protein (TBP; TaqMan endogenous control) according to the manufacturer's instructions. Calculation of signals was done as suggested in the PE Biosystems Sequence Detector User Bulletin and elsewhere [21]. Briefly, threshold cycle (Ct) is defined as the first fluorescent signal reaching statistical significance above background. For each individual condition, normalisation to Ct values of the house keeping gene (TBP) was performed (calculation of ΔCt values). Subsequently, relative expression levels were evaluated by using transcript levels of unstimulated controls of day 3 as calibrator (ΔΔCt).

### Protein extraction and Western blot analyses

Isolation of total cellular protein was performed by freezing (liquid nitrogen)-thawing in a buffer containing 50 mM Tris-HCl, 125 mM NaCl, 0,1% NP-40, 5 mM EDTA, 50 mM NaF, 1 mM DTT, protease inhibitor cocktail 1:100 and HALT Phosphatase Inhibitor Cocktail 1:100 (Pierce, Rockford, IL). Nuclear/cytoplasmic extracts were prepared using NE-PER nuclear and cytoplasmic extraction reagent according to the manufacturer's instructions (Pierce, Rockford, IL). For detection of PR or FOXO1A protein expression equal amounts (20 μg) of total or nuclear protein measured by Bradford assay (Bio-Rad Laboratories, Hercules, CA) were separated on 8.5% (PR) or 10% (FOXO1A) SDS/polyacrylamide gels. For analyses of IGFBP1 concentrations in supernatants (1 ml aspirated from 12 wells) sample preparation was performed under non-reducing conditions. Volumes were normalised to the respective cell numbers measured with Casy Cell Counter, adjusted to 20 μl with culture medium and 20 μl of 2× Novex Tris-Glycine Sample Buffer (Invitrogen, Carlsbad, CA) were added. After incubation for 10 minutes at room temperature, samples were loaded onto 12.5% SDS/polyacrylamide gels. After separation proteins were transferred onto methanol-activated polyvinyliden difluoride (PVDF) membranes (Amersham, Buckinghamshire, UK) in a buffer containing 25 mM Tris.Cl, pH 8.3, 0,5% SDS, 192 mM glycine, 20% methanol. Subsequently PVDF membranes were blocked with 5% non-fat dry milk/Tris-buffered Saline-Tween 20 (TBS-T) for 60 minutes at room temperature to eliminate unspecific binding and incubated overnight with primary antibodies in 0.5% non-fat dry milk/TBS-T at 4°C as described [21]. The following primary antibodies were used: mouse anti-human IGFBP1 (1 μg/ml clone 33627; R&D Systems, Minneapolis, MN); rabbit anti-human PR (1:1000; Cell Signaling Technology); rabbit anti-human FOXO1A (1:1000, C29H4, Cell Signaling Technology). PVDF membranes were washed three times for 5 minutes in 1× TBS-T and subsequently treated with secondary anti-mouse or anti-rabbit horseradish peroxidase-linked antibodies (anti-mouse IgG, Sigma; anti-rabbit IgG, Amersham; both diluted 1:50.000 in 0.5% non-fat dry milk/TBS-T). After one hour of incubation at room temperature blots were rinsed three times for 5 minutes and signals were developed by using ECL Plus Western blotting detection system according to the manufacturer's instruction (Amersham). Finally membranes were exposed to autoradiography for visualization of signals. Antibodies binding IGFBP1, FOXO1A, PR-A and PR-B detected specific signals at 32 kD, 78 kD, 90 kD, and 118 kD, respectively. For positive control of PR protein expression total protein lysates of the breast cancer cell line T47D were utilised. To monitor equal protein loading of cellular extracts, blots were again washed three times with 1xTBS-T for 10 minutes, blocked in 5% non-fat dry milk for 60 minutes and incubated with rabbit anti- human GAPDH antibody (1:1000; Cell Signaling Technology) or mouse anti-human TopoIIβ antibodies (0.25 mg/ml; BD Biosciences, normalization of nuclear protein extracts) over night at 4°C. GAPDH (37 kD) was detectable both in cytoplasmic and nuclear fractions as recently shown [22], whereas TopoIIβ (180 kD) was exclusively found in the nucleus.

### Prolactin ELISA

Secreted PRL was measured in culture supernatants using a PRL ELISA according to the manufacturer's instructions (Alpco, Salem, NH) and normalised to cell numbers. Intra-assay precision levels indicated by the coefficient to variation ranged between 2.1% and 4.6% and inter-assay precision levels between 3.1% and 5.4%; sensitivity was 2 ng/ml.

### Statistical analysis

For comparison of two conditions of interest statistical analyses were performed with Student's paired T-test or Mann-Whitney U test using SPSS 14 (SPSS Inc., Chicago, IL). Gaussian distribution and equality of variances were examined with Kolmogorov-Smirnov test and Levene's test, respectively. A T-test p-value of < 0.05 was considered statistically significant.

## Results

### Morphological characterization and proliferation of HDSC and THESC upon in vitro differentiation

**Figure 1:**
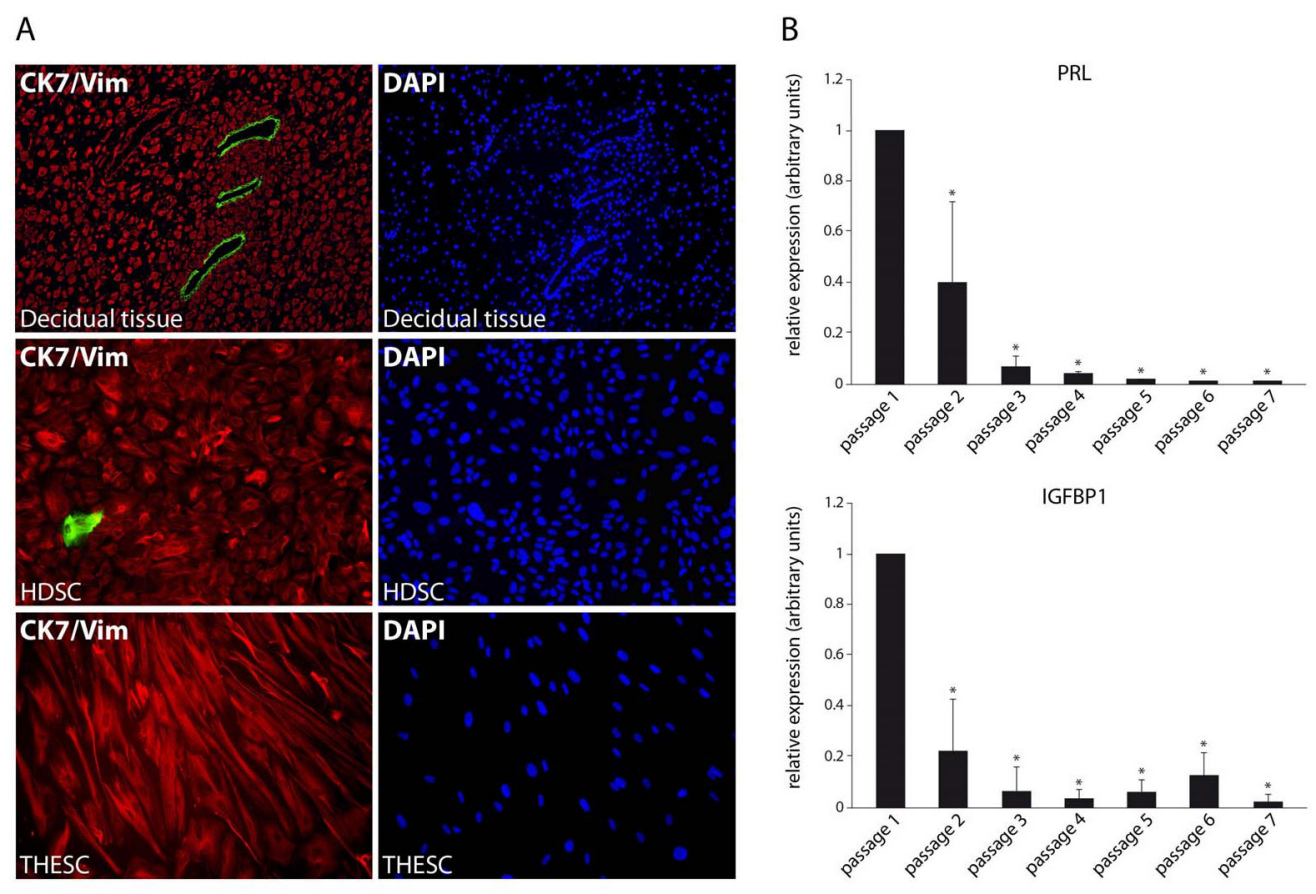
**Characterization of first trimester decidual tissue, primary HDSC and THESC using immunofluorescence and quantitative real-time PCR**. Preparation of tissue sections, isolation of primary HDSC, cultivation of THESC and quantitative real-time PCR were performed as described in Materials and Methods. **A) **Immunofluorescence of decidual cells and tissue. First trimester decidual tissue isolated at 10^th ^weeks of pregnancy, primary human decidual stromal cells at passage 3, and THESC are depicted. Double immunofluorescence staining with antibodies recognizing vimentin (red) and cytokeratin 7 (green) to detect stromal or epithelial cells, respectively, are shown. The respective counterstainings of tissues and cultures with DAPI are depicted on the right hand side. Representative pictures were taken at a 100-fold (decidual tissue) and 200-fold (cultivated cells) magnification, respectively. **B) **Quantitative real-time PCR measuring basal PRL and IGFBP1 mRNA expression in HDSC in a passage-dependent manner. For normalization values at passage 1 were arbitrarily set at 1. Bars represent mean values ± S.D. of four different experiments performed in duplicates. * indicates p < 0.05 compared to passage 1.

**Figure 2:**
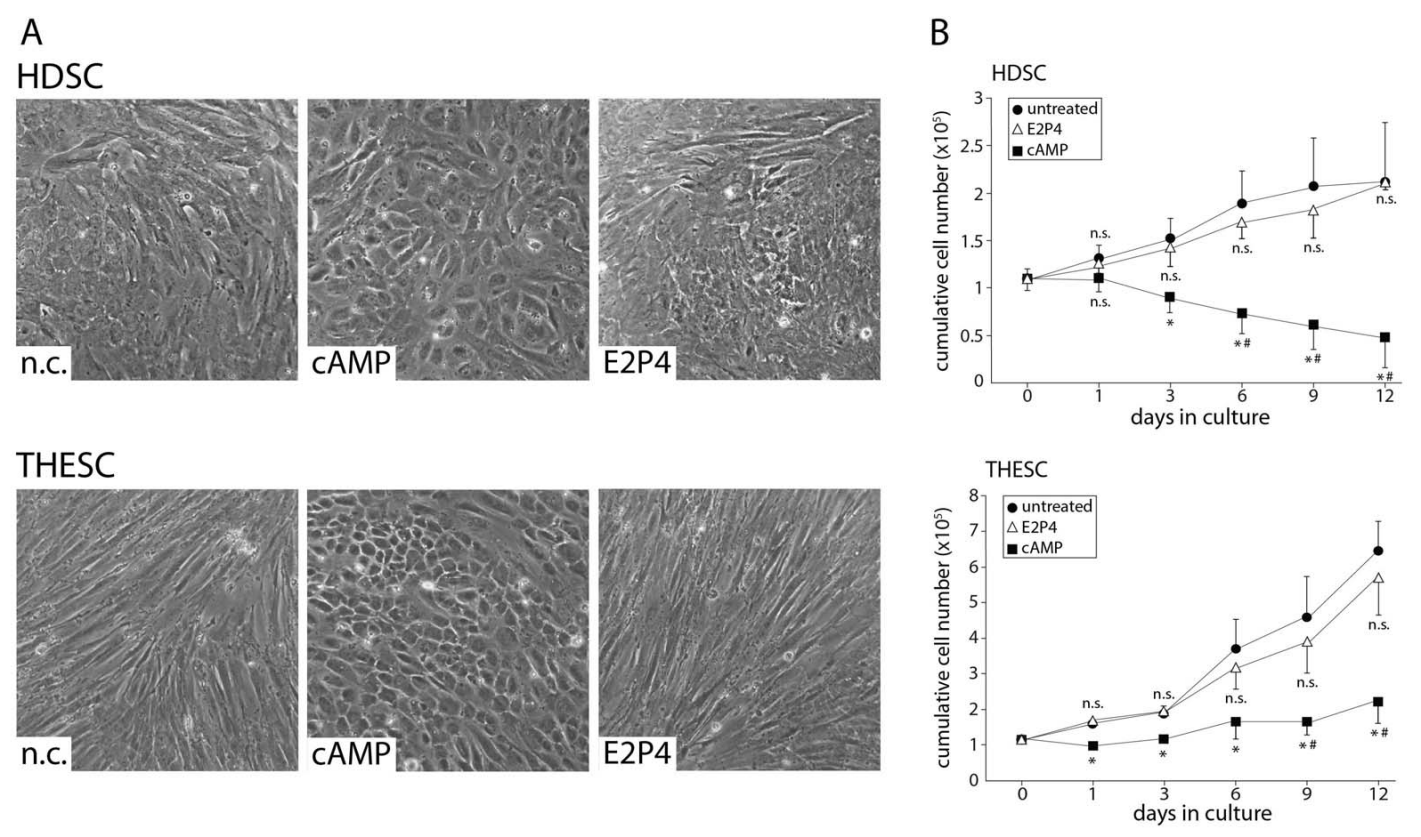
**Morphological differentiation and proliferation of HDSC and THESC in the absence or presence of cAMP or E2P4**. Cultivation, in vitro decidualization and proliferation assays of HDSC and THESC were performed as described above. **A) **Morphological characteristics of untreated and cAMP or E2P4-stimulated HDSC and THESC at day 6 of in vitro differentiation. Representative pictures at a 100-fold magnification are shown. **B) **Proliferation of HDSC and THESC in the absence or presence of cAMP or E2P4. Cumulative cell numbers of each cultures system (n = 4) were measured in duplicates at 1, 3, 6, 9, and 12 days of in vitro decidualization. * indicates p < 0.05 compared to negative control (n.c.) of the same day; # indicates p < 0.05 compared to n.c. of day 0; n.s., not significant compared to n.c. of the same day.

Decidual tissue attached to first trimester placentae, purified HDSC and THESC were analysed with respect to epithelial and stromal cell content using immunofluorescence (Figure 1A). Sections of embedded, decidual tissue showed vimentin and cytokeratin 7 expressions in stromal cells and residual glands, respectively. Primary HDSC characterized at passage 3 contained mostly vimentin-positive cells with a small numbers of cytokeratin 7-positive, contaminating epithelial cells (0.83% ± 0.38 S.D., n = 6) whereas 100% of THESC expressed vimentin. Since HDSC were derived from early pregnancy decidua, cultures might already express elevated levels of decidua-specific proteins. Hence, to determine the optimal time point for in vitro re-decidualization basal mRNA expression of decidual marker genes was analysed in a passage-dependent manner. Quantitative real-time PCR revealed residual PRL and IGFBP1 mRNA levels in the early passages 1 and 2 which dropped upon further cultivation (Figure 1B). Therefore, in vitro differentiation was routinely performed with cultures at passage 3 to 5. Subsequently, morphological changes and proliferation of in vitro decidualizing cultures were assessed (Figure 2). Compared to THESC showing a spindle-shaped, fibroblastoid morphology, areas with cells displaying a cobblestone-like morphology were observed in untreated HDSC (Figure 2A). Stimulation with cAMP strongly induced the polygonal phenotype (Figure 2A) and provoked growth arrest in both cell types (Figure 2B). Long-term treatment with cAMP additionally reduced viability of HDSC since compared to controls cell numbers significantly decreased from day 6 onwards. In contrast, loss of cell numbers could not be observed in cAMP-treated THESC. Stimulation with E2P4 did not induce morphological changes in HDSC or THESC (Figure 2A). Proliferation of both cell types was not affected upon hormone treatment (Figure 2B).

### Differentiation-dependent expression of progesterone receptors in HDSC and THESC

**Figure 3:**
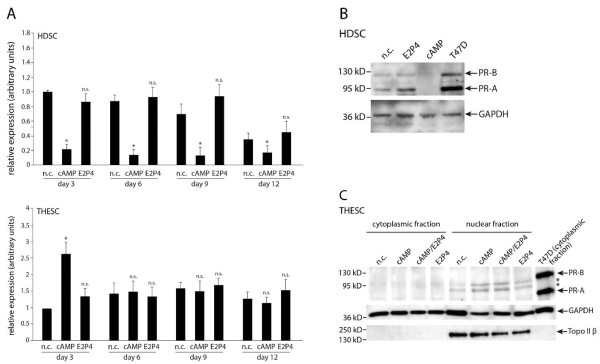
**Expression analyses of PR in differentiating HDSC and THESC**. Quantitative **r**eal-time PCR and Western blot analyses were performed as described in Materials and Methods. Representative examples of Western blots are shown. **A) **PR mRNA expression at day 3, 6, 9, and 12 of cAMP or E2P4 stimulation. For relative quantification of total PR mRNA expression, n.c. of day three was arbitrarily set at 1 (calibrator). Bars indicate mean values ± SEM of seven and five different experiments performed in HDSC and THESC, respectively. PCR reactions were done in duplicates. * indicates p < 0.05 compared to the n.c. of the same day; n.s., not significant. **B) **Western blot analyses showing PR protein expression in total cellular extracts of dezidualizing HDSC. Specific signals detecting PR-A (90 kD) and PR-B (118 kD) in the absence or presence of cAMP or E2P4 (day 9) are depicted. Total extracts prepared from T47D cells were used as a positive control. Marker bands are indicated on the left. GAPDH was used as a loading control. **C) **Western blot analyses of cytoplasmic and nuclear extracts isolated from unstimulated and differentiated THESC which had been treated with cAMP and/or E2P4 for 9 days. Specific signals detecting PR-A (90 kD) and PR-B (118 kD) in T47D are depicted. Marker bands are indicated on the left and unspecific signals are indicated by stars. GAPDH and TopoIIβ were used as loading controls for cytoplasmic and nuclear extracts, respectively.

Expression of PR was analysed in cAMP and/or E2P4 treated HDSC and THESC (Figure 3). Quantitative real-time PCR revealed that cAMP significantly decreased PR mRNA in HDSC at the different time points, whereas E2P4 did not affect transcript levels (Figure 3A). Surprisingly, considerable, constitutive expression of PR mRNA was detectable in THESC, but in contrast to HDSC cAMP did not alter transcript levels (Figure 3A). Western blot analyses showed that HDSC also produced the PR proteins, PR-A (90 kD) and PR-B (118 kD), which were identified by using the PR-expressing breast cancer cell line T47D [23] as a positive control (Figure 3B). Both isoforms were suppressed in the presence of cAMP (day 9 of differentiation), but did not change upon E2P4 treatment. However, the PR antibody specifically recognizing PR-A and PR-B in HDSC and T47D cells failed to detect correct signals in cytoplasmic and nuclear extracts of THESC in the absence or presence of cAMP and/or E2P4 (Figure 3C).

### Expression of decidual marker genes in differentiating HDSC and THESC

**Figure 4:**
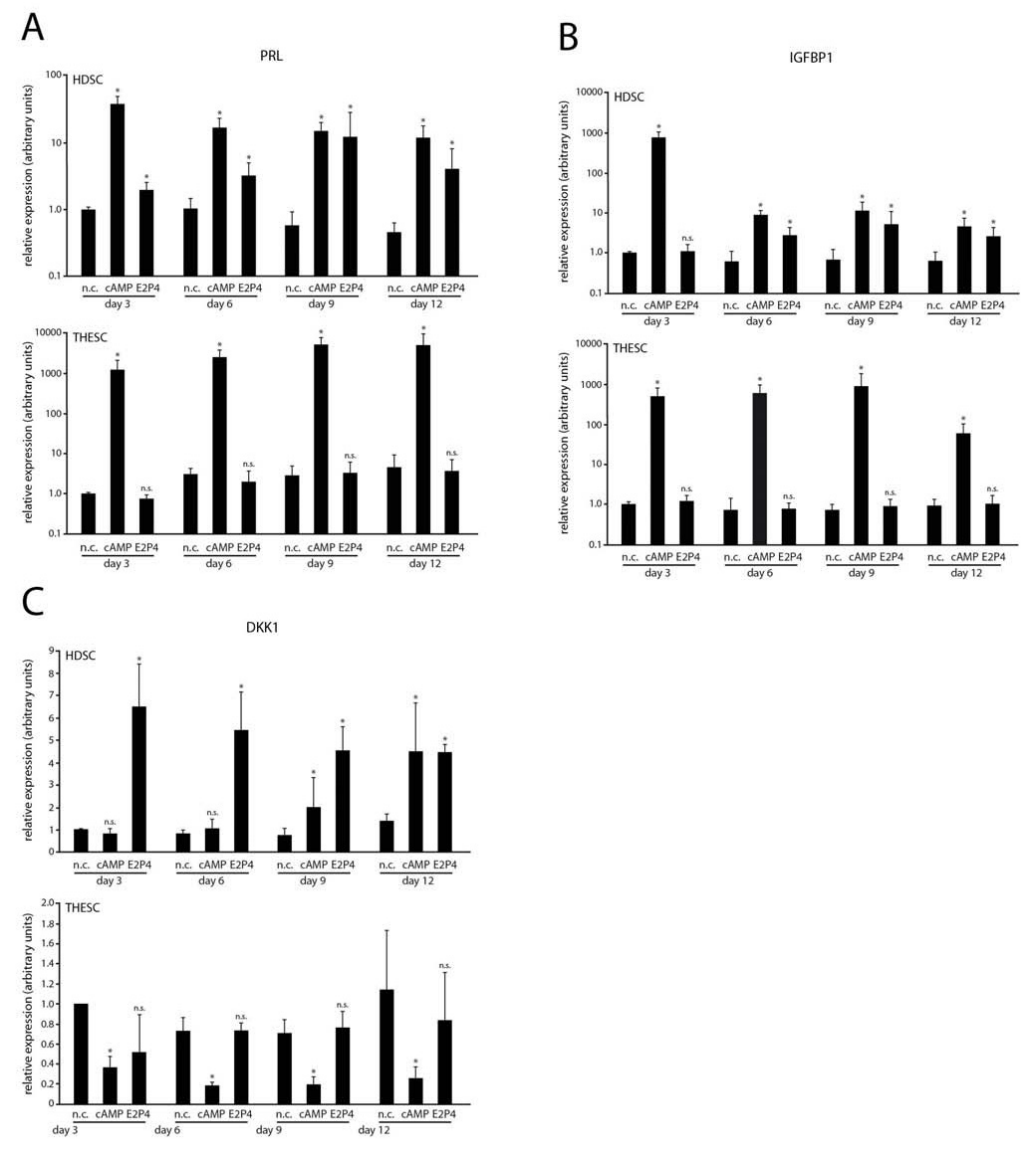
**Quantitative real-time PCR measuring mRNA expression of decidual marker genes in differentiating HDSC and THESC**. Cultures were incubated with cAMP or E2P4 for 3, 6, 9 and 12 days. Cells without stimuli were kept in parallel representing non-stimulated controls (n.c.). RNA extraction and quantitative real-time PCR were performed as described in Methods. For relative quantification of mRNA expression n.c. of day 3 was arbitrarily set at 1 (calibrator). Bars indicate mean values ± SEM of seven (HDSC) and five (THESC) different experiments/PCR reactions performed in duplicates.* depicts p < 0.05 compared to the n.c. of the same day; n.s., not significant. **A) **PRL mRNA expression. **B) **IGFBP1 mRNA expression. **C) **DKK1 mRNA expression.

Next, expression of decidual marker genes was analysed in untreated cultures and upon differentiation in the presence of cAMP or E2P4 using quantitative real-time PCR (Figure 4). PRL mRNA (Figure 4A) and IGFBP1 mRNA (Figure 4B) were strongly induced in HDSC upon cAMP treatment. However, this response was transient and highest levels of PRL and IGFBP1 transcripts were detected at 3 days of cAMP stimulation. Similarly, PRL mRNA (Figure 4A) and IGFBP1 mRNA (Figure 4B) were induced in cAMP-treated THESC, but expression of both genes remained at high levels during differentiation. E2P4 treatment of HDSC provoked a steady increase of transcript levels of PRL (Figure 4A) and IGFBP1 (Figure 4B) which was significantly different to controls from day 3 (PRL) and day 6 (IGFBP1) onwards. In contrast neither PRL (Figure 4A) nor IGFBP1 (Figure 4B) mRNA were induced in THESC in the presence of E2P4. DKK1 has been recently described as a progesterone-dependent but cAMP-independent gene in decidualization [24]. Accordingly, E2P4 increased DKK1 mRNA expression in HDSC at day 3, 6, 9, and 12 of in vitro differentiation (Figure 4C). Compared to the respective controls, its expression was also induced upon cAMP treatment at 9 and 12 days of HDSC differentiation (Figure 4C). In THESC, however, DKK1 mRNA was not altered and suppressed in the presence of E2P4 and cAMP, respectively (Figure 4C).

**Figure 5:**
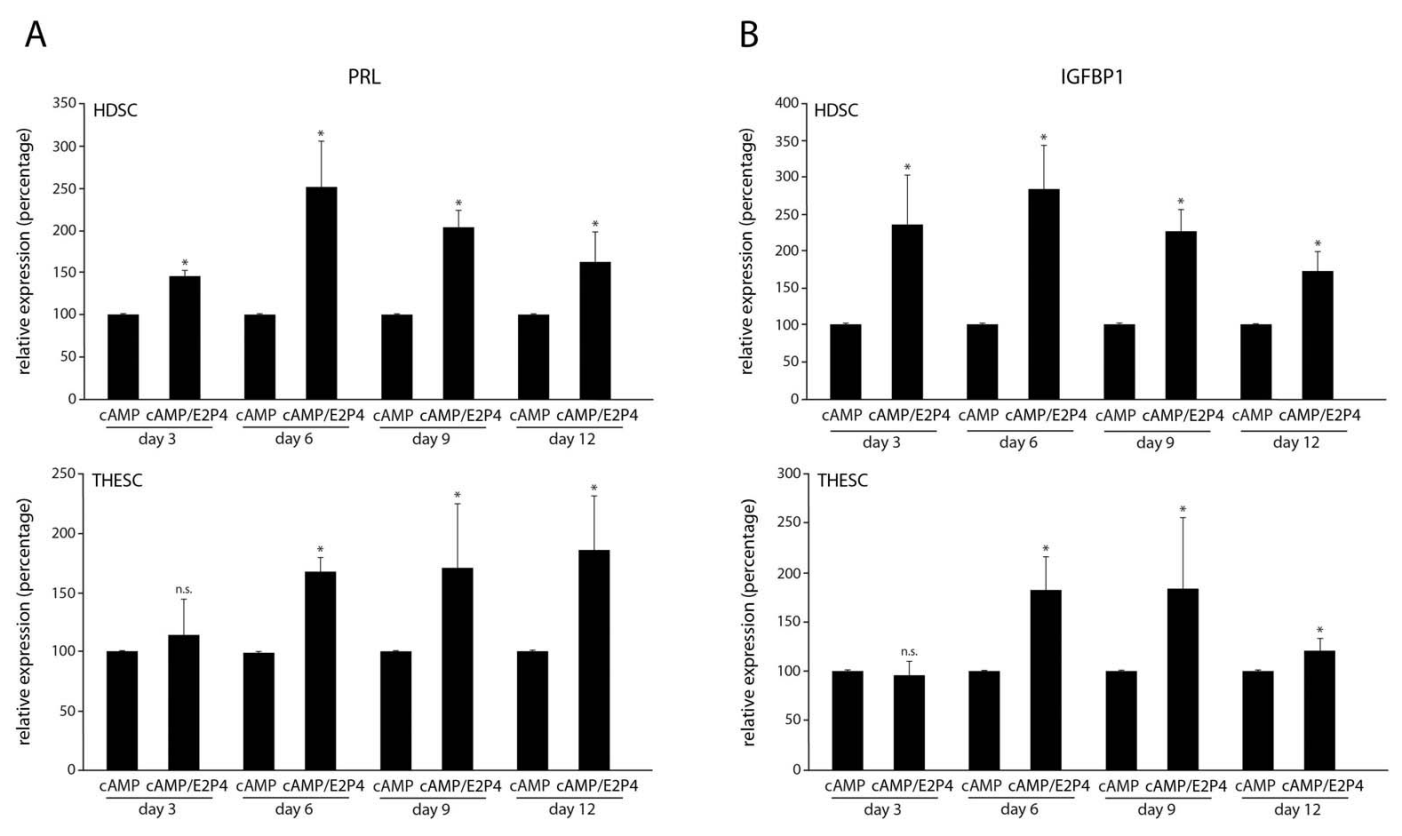
**Quantitative mRNA expression of decidual marker genes upon cAMP stimulation or combined treatment with cAMP and E2P4 in differentiating HDSC and THESC**. Cultures were incubated with E2P4 and/or cAMP for for 3, 6, 9 and 12 days and transcript levels were analysed by real-time PCR. For relative quantification of mRNA expression, cAMP-treated samples were arbitrarily set at 100%. Bars indicate mean values ± SEM of five and four different experiments performed in HESC and THESC, respectively. PCR reactions were done in duplicates. * indicates p < 0.05 compared to the cAMP-stimulated sample of the same day; n.s., not significant. **A) **PRL mRNA expression. **B) **IGFBP1 mRNA expression.

Moreover, the effects of a combined treatment with cAMP and E2P4 were analysed in HDSC and THESC (Figure 5). Real-time PCR of PRL (Figure 5A) and IGFBP1 (Figure 5B) mRNAs revealed that, compared to cAMP treatment alone, co-stimulation with cAMP and E2P4 further enhanced mRNA expression in HDSC. Increased mRNA expression of PRL (Figure 5A) and IGFBP1 (Figure 5B) was also detectable in THESC upon cAMP/E2P4 co-stimulation although the effects were less pronounced than in HDSC.

**Figure 6:**
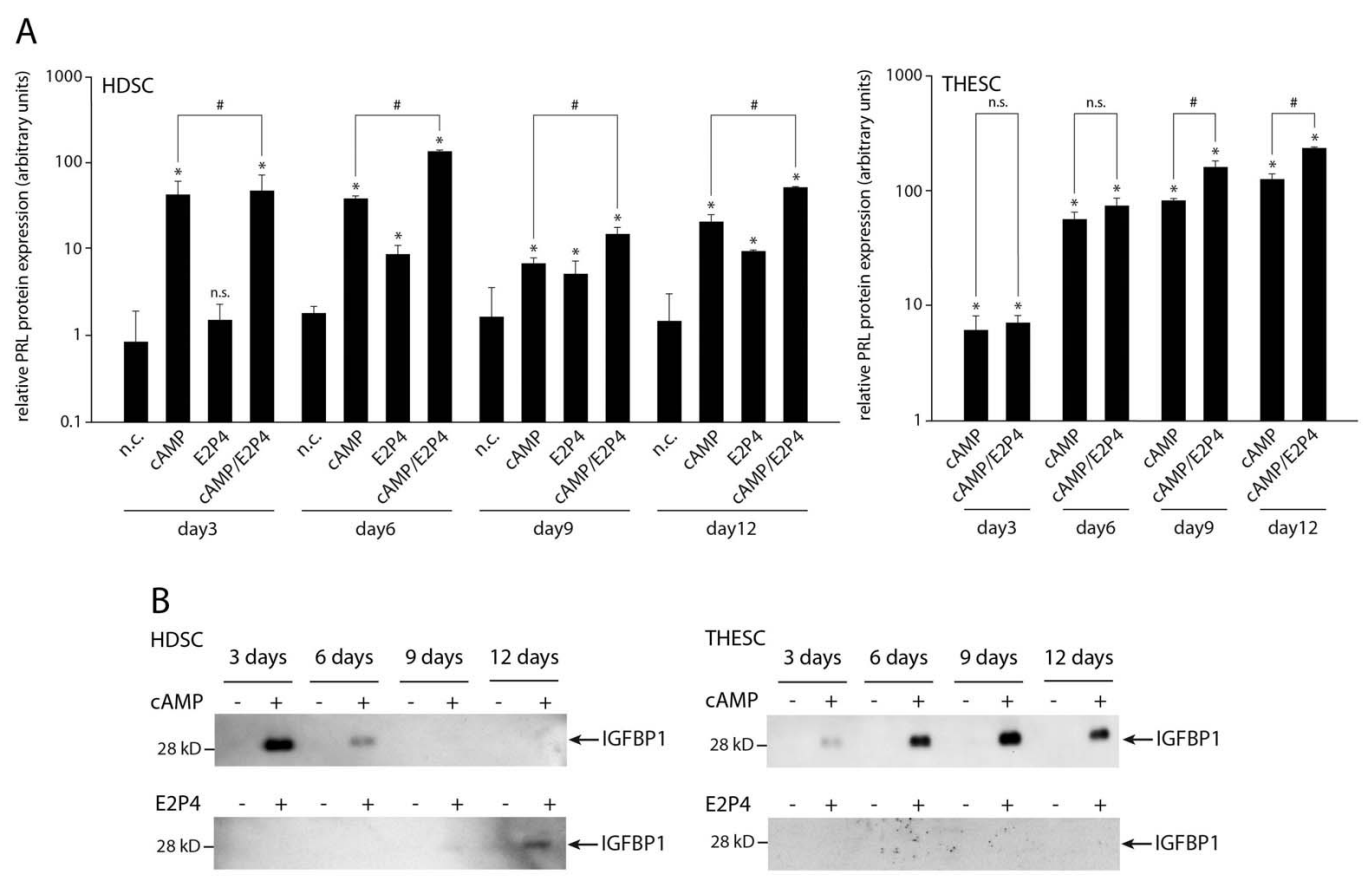
**Differentiation-dependent protein expression of PRL and IGFBP1 in culture supernatants of HDSC and THESC**. Cells were stimulated in the absence or presence of cAMP and/or E2P4 for 3, 6, 9 and 12 days. Supernatants were collected and PRL and IGFBP1 protein concentrations were measured by ELISA and Western blotting, respectively. Normalization of protein concentrations, ELISA, and immunodetection of IGFBP1 on membranes were performed as mentioned in Materials and Methods. **A) **ELISA of PRL in HDSC and THESC. Data represent mean values ± SEM of three experiments performed in duplicates. * indicates p < 0.05 compared to negative control (n.c.) of the same day; n.s., not significant; # indicates p < 0.05 between cAMP-treated and cAMP/E2P4-stimulated cultures. PRL expression in controls and E2P4-treated THESC were below the detection limit and therefore omitted from the graph. **B) **Western blot showing IGFBP1 (32 kD) expression in HDSC and THESC. Marker bands (kD) are depicted on the left side. Representative examples are shown.

Subsequently, PRL and IGFBP1 protein expression were analysed in supernatants of HDSC and THESC using ELISA and Western blotting, respectively (Figure 6). Similar to its mRNA levels, PRL protein was induced upon cAMP or E2P4 stimulation of HDSC and expression further increased in the combined cAMP/E2P4 treatment (Figure 6A). PRL protein was also detectable in cAMP-treated THESC (Figure 6A) but absent from controls and E2P4-treated cells (not shown). Compared to cAMP alone, its expression increased upon supplementation of cAMP/E2P4. Soluble IGFBP1 protein also closely mimicked its mRNA expression under the different stimuli. Treatment of HDSC with cAMP provoked transient expression of IGFBP1 with highest levels at day 3, whereas in the presence of E2P4 the protein was detectable at day 9 and further increased at day 12 (Figure 6B). In cAMP-treated THESC high IGFBP1 levels were observed between day 6 and day 12, whereas the particular protein was absent from controls and E2P4-treated cultures (Figure 6B).

### Differentiation-dependent expression and localization of FOXO1A in HDSC and THESC

**Figure 7:**
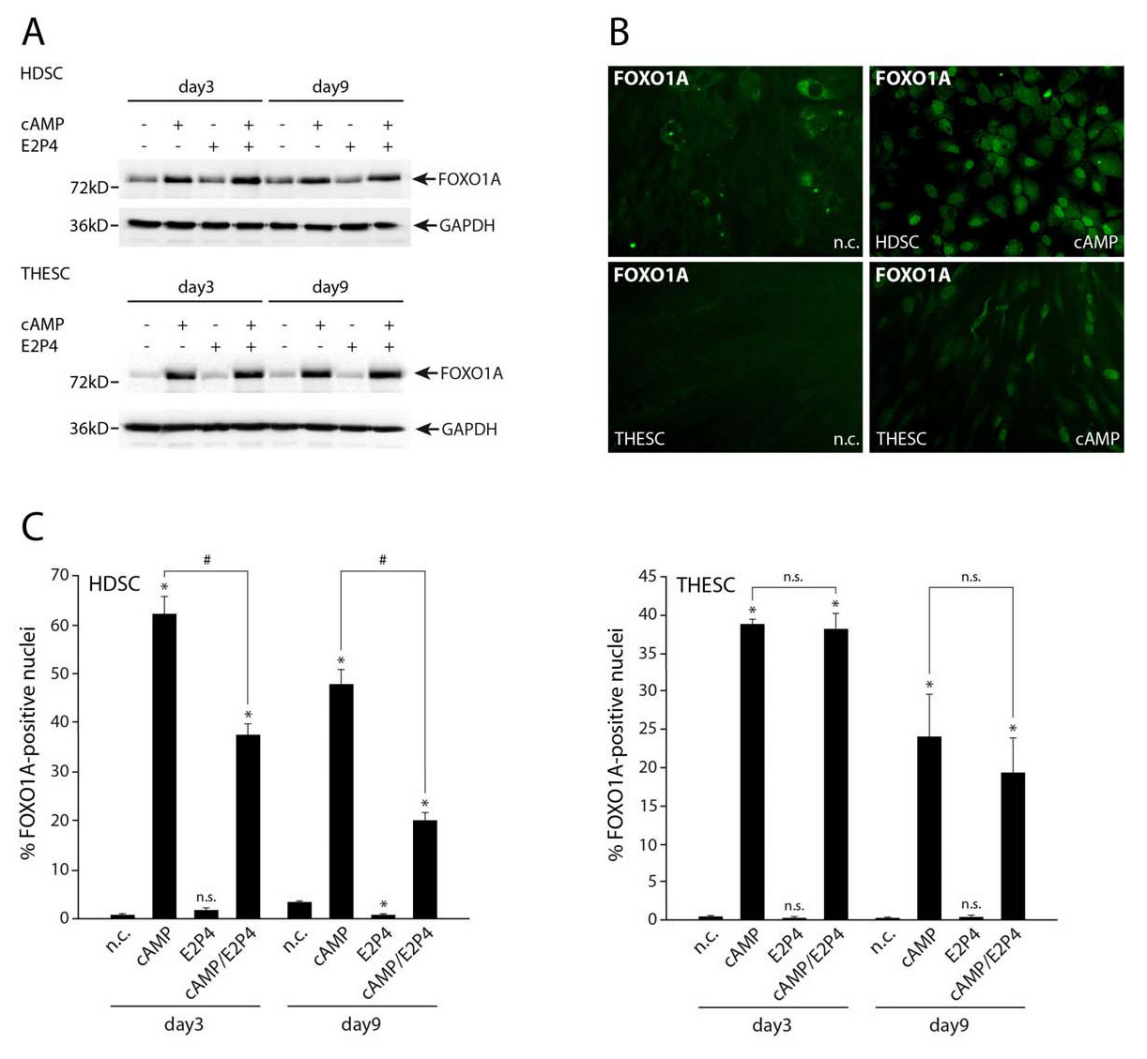
**FOXO1A protein expression during in vitro differentiation of HDSC and THESC**. Cultures were incubated with cAMP and/or E2P4 for 3 and 9 days. Protein expression and localization of FOXO1A were determined by Western blotting and immunofluorescence, respectively, as described in Materials and Methods. A) Western blot detecting FOXO1A (78 kD) in total cellular extracts. Marker bands (kD) are depicted on the left side. GAPDH (37 kD) was used as a loading control. Representative examples are shown. B) Immunflourescence in untreated and cAMP-stimulated HDCS and THESC. Representative pictures at a 200 fold magnification are shown. C) Quantification of FOXO1A-positive nuclei in cAMP and/or E2P4-treated HDSC and THESC after immunofluorescence. Ratio (percentage) of positive nuclei were evaluated as described above. Bars represent mean values ± SEM of three experiments. * indicates p < 0.05 compared to negative control (n.c.) of the same day; n.s., not significant; # indicates p < 0.05 between cAMP-treated and cAMP/E2P4-stimulated cultures.

Finally, expression and localization of FOXO1A, a critical, transcriptional regulator of the decidualization-associated genes PRL and IGFBP-1 [12], were investigated (Figure 7). Western blot analyses revealed that cAMP-increased FOXO1A protein at all time points of differentiation in HDSC and THESC, whereas E2P4 did not change expression in both culture systems (Figure 7A). Immunofluorescence of cells revealed nuclear localization of FOXO1A upon cAMP stimulation (Figure 7B). In unstimulated cultures, however, FOXO1A was diffusely distributed within the cytoplasm (HDSC) or absent (THESC). Quantification of fluorescent images indicated an increase of FOXO1A-postive nuclei in both cell types upon cAMP treatment, whereas E2P4 was not effective (Figure 7C). Compared to cAMP stimulation, however, the combined cAMP/E2P4 treatment decreased numbers of FOXO1A-postive nuclei in HDSC, but not in THESC.

## Discussion

Correct decidualization of the endometrium is indispensable for proper implantation, fetal development and successful progression of pregnancy. Hence, the underlying molecular mechanisms are critical to our understanding of implantation defects associated with infertility. Accordingly, in the recent years diverse in vivo animal models have been developed involving mouse and primate-systems [25,26]. In contrast to all other species, however, human decidualization starts spontaneously in the second half of the menstrual cycle in response to the predominating ovarian progesterone milieu. Therefore, adequate human in vitro models for decidualization had to be established and still comprise the basis to translate and verify results obtained from animal models. Indeed, HESC obtained from endometrial biopsies of pre-menopausal women or from uterine tissue after hysterectomy for benign gynaecological diseases are widely used for cAMP and/or E2P4 or MPA-induced in vitro differentiation [12,14]. However, availability of these samples might be limited, rising the need for alternative systems.

### Culture systems utilized for cAMP-dependent in vitro decidualization

Therefore, efforts have been made to immortalize human endometrial stromal cells to overcome the restricted supply of primary HESC. Various immortalized cell lines were established after lentiviral transduction of plasmids expressing either SV40 large T antigen [19,27,28] or telomerase [20,29,30]. In the presence of cAMP these cell lines were shown to differentiate into a cobblestone-like phenotype and to express marker genes of decidualization such as IGFBP1, PRL and FOXO1A [30]. The cAMP-dependent induction of PRL and IGFBP1 in THESC analysed here confirms these previous observations. Hence, it can be concluded that the cAMP-stimulated expression of markers genes might be similar in the immortalized cells and primary HESC suggesting suitability of cell lines for the particular differentiation protocol.

### The role of progesterone in in vitro decidualization

On the other hand, it was suggested that cAMP may not be sufficient for maintenance of decidua-specific gene expression in differentiating primary HESC but requires the presence of progesterone [12]. Indeed, upon cAMP treatment MPA-dependent induction of PRL has been observed in HIESC-2 and St-TIb cultures [28,30] and similar to primary HESC [12] the combined stimulation was more efficient than cAMP treatment alone [30]. Moreover, MPA-induced expression of PRL and IGFBP1 in absence of cAMP has also been noticed in immortalized cultures [20,29] suggesting a physiological, PR-dependent response. However, as has been recently pointed out MPA additionally interacts with glucocorticoid and androgen receptor [30], the latter being also involved in decidualization [31]. Therefore, a response to natural P4 rather than MPA and expression of PR as occurs in primary HESC could be regarded as a hallmark of physiological decidualization and cell identity. However, P4 responsiveness could not be detected in two different immortalized endometrial stromal cell lines [18,19]. Similarly, in THESC analysed here P4 did not affect expression of DKK1, a progesterone-inducible gene in HESC [24].

### Expression of progesterone receptors in different model systems of decidualization

Analyses of PR protein expression in the different immortalized stromal cell lines such as THESC are either lacking [20] or unclear due to the fact that appropriate positive controls are missing [28,29]. Also, specificity of PR signals of a particular cell line [28] was questioned since the PR antibody used may show unspecific cross-reactivity [32]. The Western blot analyses performed here suggest that PRs are absent from THESC providing an explanation for the absence of P4 responsiveness. Although the PR antibody used in our study recognized two weak signals in nuclear extracts of THESC, these bands showed different molecular sizes than PR-A and PR-B produced in T47D cells. Differently to the regulation of PR in HDSC and primary HESC [13] the signals were not suppressed upon cAMP-induced differentiation. Therefore, we conclude that the antibody unspecifically recognizes a nuclear protein in THESC, particularly in the absence of PR protein. Interestingly THESC, like the previously described St-TIb cells [30], expressed considerable amounts of PR mRNA despite the absence of PR proteins. Moreover, combined treatment with cAMP and P4 of St-TIb [30] or of THESC provoked elevated PRL mRNA and protein expression compared to cAMP treatment alone. Considering the absence of nuclear PR protein in both cell lines activation through P4 remains elusive. Eventually, the effect might be explained by the non-genomic actions of progesterone [33].

### Human decidual stromal cells as an alternative model for in vitro decidualization

An alternative for studying endometrial differentiation in untransformed cultures has been suggested by using isolated stromal cells from human term deciduas [34]. MPA- and cAMP-dependent induction of PRL and IGFBP1 was observed in these cultures reflecting the expression patterns observed in primary HESC. Furthermore, due to the strong similarities between HESC and term decidual stromal cells it was suggested that both cell types could be precursor decidual cells arising from the same cell lineage [34]. In the present study primary HDSC obtained from decidual tissue of early pregnancy terminations have been evaluated as a model system for in vitro differentiation. HDSC of early gestation develop in a progesterone-rich environment promoting differentiation. Indeed, at the time of isolation elevated basal levels of PRL and IGFBP1 were noticed which upon in vitro cultivation decreased in a passage-dependent manner. After a minimum of 3 passages basal marker gene expression of unstimulated cultures has sufficiently decreased to commence re-decidualization. Indeed, differentiated HESC also lose expression of decidua-specific genes upon withdrawal of stimuli [12]. Hence, we conclude that the absence of cAMP and progesterone promotes de-differentiation of HDSC, although the presence of residual progenitor cells in isolated cultures cannot be excluded. Indeed, recent evidence suggested that first trimester decidua contains spindle-shaped, multipotent decidual stromal cells capable of differentiating into diverse cell types including PRL-producing, differentiated decidual cells [35].

### Utilization of HDSC for cAMP or E2P4-induced in vitro decidualization

Several lines of evidence presented here suggest that HDSC could provide an alternative model system to investigate cAMP and/or E2P4-dependent decidualization. In accordance with the published literature using HESC as model systems, HDSC had to be confluently seeded for proper differentiation. Experiments with cells at lower density revealed delayed induction of marker gene expression when stimulated with cAMP or E2P4 (unpublished observation). Despite slow proliferation due to contact inhibition, untreated cultures did not spontaneously differentiate as verified by constant basal levels of PRL and IGFBP1 mRNA during the entire stimulation period. The presence of cAMP, provoking changes in phenotype as well as growth arrest, resulted in rapid induction of marker genes of decidualization, i.e. PRL, IGFBP1 and FOXO1A. However, PRL and IGFBP1 mRNA and protein were transiently expressed and decreased at later time points of cAMP-stimulated differentiation. Similar observations have been published regarding PRL and IGFBP1 secretion in HESC [12,13,36]. Hence, we assume that similar to HESC cAMP treatment alone may not be sufficient for maintenance of decidual marker gene expression in HDSC.

In contrast to cAMP, administration of E2P4 did not morphologically change HDSC but provoked a continuous increase of PRL and IGFBP1 transcript and protein levels. Indeed, it has been published that progestins also induce a steady but modest increase of PRL and IGFBP1 mRNA expression in HESC [37]. Interestingly, E2P4- and cAMP-induced PRL protein levels were similar at day 9 of HDSC differentiation. In contrast, MPA-induced PRL concentrations at day 6 and 8 of HESC differentiation were shown to be approximately 1000 fold less than cAMP-induced levels [12,13]. From these observations we conclude that E2P4-dependent in vitro decidualization could be more efficient in HDSC than in HESC. This might be caused by higher endogenous levels of cAMP in untreated HDSC or a steeper increase in cAMP concentrations upon E2P4 treatment [15]. On the other hand cAMP, known to induce apoptosis in different culture systems [38,39], provoked loss of HDSC after 6 days of incubation. Therefore, elevated endogenous cAMP levels in HDSC as compared to HESC could also provide an explanation for the higher sensitivity of these cells to the cell-damaging effects of exogenously added 8-Bromo-cAMP.

### Combined cAMP and E2P4 treatment of HDSC

Compared to cAMP stimulation alone the combined treatment with cAMP and E2P4 further increased PRL and IGFBP1 mRNA expression and PRL protein levels in HDSC. Elevated expression of these genes might suggest that the combined treatment is necessary for complete and sustained marker expression and differentiation of HDSC as has been previously suggested for HESC [12,13].

Along those lines, a natural progesterone response could be detected in HDSC since P4 increased mRNA levels of DKK1, which had been previously identified as a progesterone-inducible gene [24], at early and late time points of differentiation. In contrast to THESC, protein expression of PR-A and PR-B was detectable in isolated HDSC and both isoforms were downregulated upon cAMP treatment. These results are in agreement with studies performed in primary HESC triggered into differentiation with the particular stimulus [13]. Furthermore, PR-A is the dominant isoform expressed in HDSC mirroring the expression pattern observed both in human endometrium during the secretory phase *in vivo *as well as in isolated HESC in vitro [13,40,41]. Finally, compared to cAMP stimulation alone, combined treatment with cAMP and E2P4 decreased nuclear abundance of FOXO1A in HDSC indicating a hormone-dependent effect. These data are agreement with previous observations in HESC [42] suggesting that progesterone could also promote survival of HDSC by reducing cAMP-dependent apoptosis mediated through FOXO1A. Genomic actions of nuclear PRs likely play a role in cytoplasmic retention of FOXO1A since this phenomenon could not be observed in PR-deficient THESC.

## Conclusion

Taken together, primary HDSC which were isolated from first trimester decidual tissue and treated with cAMP and/or E2P4 exhibit all features of correct decidualization known from studies performed with HESC as a model system. Even though single administration of cAMP longer than 6 days could not be recommended due to cell loss, short term differentiation proceeded correctly and mirrored expression patterns in HESC. E2P4 stimulation provoked continuous increases in marker gene expression suggesting that hormone-treatment of HDSC provides a suitable in vitro system to study human decidualization. Beside similar properties of HESC and HDSC upon differentiation, the usage of first trimester decidual tissues may eventually overcome the problem of limited availability of endometrial samples which can only be obtained when a benign gynaecological disease has been diagnosed. Regarding THESC as a model system cAMP-induced morphological transformation and marker gene expression can be achieved. However, P4-dependent differentiation may not be applicable due to the lack of physiological progesterone-responsiveness and the absence of classical PRs.

## List of abbreviations

C/EPBβ: CAAT enhancer binding protein β; cAMP: adenosine 3',5'-cyclic monophosphate; DKK1: Dickkopf-1; E2: 17β-estradiol-acetate; ECM: extracellular matrix; ER: estrogen receptor; HESC: human endometrial stromal cells; HDSC: human decidual stromal cells; HUVEC: human umbilical vein endothelial cells; FOXO1A: forkhead box O1A; IGFBP1: insulin-like growth factor binding protein 1; IL: interleukin; HOX: homeobox; MPA: medroxyprogesterone acetate; NK: natural killer; P4: 4-pregnene-3,20-dione; PBS: phosphate buffered saline; PRL: prolactin; PR: progesterone receptor; Stat: signal transducer and activator of transcription; THESC: telomerase-transformed endometrial stromal cells.

## Competing interests

The authors declare that they have no competing interests.

## Authors' contributions

LS and GO performed all experiments presented in the manuscript such as primary cell culture, Western blot analyses and quantitative real-time PCR. JP helped with preparation of figures and draft on the manuscript. CF was responsible for isolation and delivery of first trimester decidual tissue. MK designed and coordinated the study and wrote the manuscript. All authors read and approved the final manuscript.

## References

[B1] Daly DC Maslar IA Riddick DH Prolactin production during in vitro decidualization of proliferative endometrium Am J Obstet Gynecol 1983 145 672 678 6829654 10.1016/0002-9378(83)90572-0

[B2] Lockwood CJ Nemerson Y Guller S Krikun G Alvarez M Hausknecht V Gurpide E Schatz F Progestational regulation of human endometrial stromal cell tissue factor expression during decidualization J Clin Endocrinol Metab 1993 76 231 236 10.1210/jc.76.1.231 8421090

[B3] Giudice LC Dsupin BA Irwin JC Steroid and peptide regulation of insulin-like growth factor-binding proteins secreted by human endometrial stromal cells is dependent on stromal differentiation J Clin Endocrinol Metab 1992 75 1235 1241 10.1210/jc.75.5.1235 1385468

[B4] Dunn CL Critchley HO Kelly RW IL-15 regulation in human endometrial stromal cells J Clin Endocrinol Metab 2002 87 1898 1901 10.1210/jc.87.4.1898 11932337

[B5] Karpovich N Klemmt P Hwang JH McVeigh JE Heath JK Barlow DH Mardon HJ The production of interleukin-11 and decidualization are compromised in endometrial stromal cells derived from patients with infertility J Clin Endocrinol Metab 2005 90 1607 1612 15613426 10.1210/jc.2004-0868 PMC1626577

[B6] Gellersen B Brosens IA Brosens JJ Decidualization of the human endometrium: mechanisms, functions, and clinical perspectives Semin Reprod Med 2007 25 445 453 10.1055/s-2007-991042 17960529

[B7] Lash GE Robson SC Bulmer JN Review: Functional role of uterine natural killer (uNK) cells in human early pregnancy decidua Placenta 2010 31 Suppl S87 92 20061017 10.1016/j.placenta.2009.12.022

[B8] Ramathal CY Bagchi IC Taylor RN Bagchi MK Endometrial decidualization: of mice and men Semin Reprod Med 2010 28 17 26 10.1055/s-0029-1242989 20104425 PMC3095443

[B9] Kim JJ Jaffe RC Fazleabas AT Blastocyst invasion and the stromal response in primates Hum Reprod 1999 14 Suppl 2 45 55 10690800 10.1093/humrep/14.suppl_2.45

[B10] Kimatrai M Oliver C Abadia-Molina AC Garcia-Pacheco JM Olivares EG Contractile activity of human decidual stromal cells J Clin Endocrinol Metab 2003 88 844 849 10.1210/jc.2002-021224 12574222

[B11] Lim HJ Wang H Uterine disorders and pregnancy complications: insights from mouse models J Clin Invest 2010 120 1004 1015 10.1172/JCI41210 20364098 PMC2846054

[B12] Gellersen B Brosens J Cyclic AMP and progesterone receptor cross-talk in human endometrium: a decidualizing affair J Endocrinol 2003 178 357 372 10.1677/joe.0.1780357 12967329

[B13] Brosens JJ Hayashi N White JO Progesterone receptor regulates decidual prolactin expression in differentiating human endometrial stromal cells Endocrinology 1999 140 4809 4820 10.1210/en.140.10.4809 10499541

[B14] Brosens JJ Gellersen B Death or survival--progesterone-dependent cell fate decisions in the human endometrial stroma J Mol Endocrinol 2006 36 389 398 10.1677/jme.1.02060 16720711

[B15] Brar AK Frank GR Kessler CA Cedars MI Handwerger S Progesterone-dependent decidualization of the human endometrium is mediated by cAMP Endocrine 1997 6 301 307 10.1007/BF02820507 9368687

[B16] Gellersen B Reimann K Samalecos A Aupers S Bamberger AM Invasiveness of human endometrial stromal cells is promoted by decidualization and by trophoblast-derived signals Hum Reprod 2010 25 862 873 10.1093/humrep/dep468 20118488

[B17] Menkhorst E Salamonsen LA Zhang J Harrison CA Gu J Dimitriadis E Interleukin 11 and activin A synergise to regulate progesterone-induced but not cAMP-induced decidualization J Reprod Immunol 2010 84 124 132 10.1016/j.jri.2009.12.001 20074812

[B18] Brosens JJ Takeda S Acevedo CH Lewis MP Kirby PL Symes EK Krausz T Purohit A Gellersen B White JO Human endometrial fibroblasts immortalized by simian virus 40 large T antigen differentiate in response to a decidualization stimulus Endocrinology 1996 137 2225 2231 10.1210/en.137.6.2225 8641169

[B19] Tamura K Yoshie M Hara T Isaka K Kogo H Involvement of stathmin in proliferation and differentiation of immortalized human endometrial stromal cells J Reprod Dev 2007 53 525 533 10.1262/jrd.18129 17272923

[B20] Krikun G Mor G Alvero A Guller S Schatz F Sapi E Rahman M Caze R Qumsiyeh M Lockwood CJ A novel immortalized human endometrial stromal cell line with normal progestational response Endocrinology 2004 145 2291 2296 10.1210/en.2003-1606 14726435

[B21] Huber AV Saleh L Prast J Haslinger P Knofler M Human chorionic gonadotrophin attenuates NF-kappaB activation and cytokine expression of endometriotic stromal cells Mol Hum Reprod 2007 13 595 604 10.1093/molehr/gam032 17525069

[B22] Sirover MA New nuclear functions of the glycolytic protein, glyceraldehyde-3-phosphate dehydrogenase, in mammalian cells J Cell Biochem 2005 95 45 52 10.1002/jcb.20399 15770658

[B23] Clemm DL Sherman L Boonyaratanakornkit V Schrader WT Weigel NL Edwards DP Differential hormone-dependent phosphorylation of progesterone receptor A and B forms revealed by a phosphoserine site-specific monoclonal antibody Mol Endocrinol 2000 14 52 65 10.1210/me.14.1.52 10628747

[B24] Tulac S Overgaard MT Hamilton AE Jumbe NL Suchanek E Giudice LC Dickkopf-1, an inhibitor of Wnt signaling, is regulated by progesterone in human endometrial stromal cells J Clin Endocrinol Metab 2006 91 1453 1461 10.1210/jc.2005-0769 16449346

[B25] Masuda H Maruyama T Hiratsu E Yamane J Iwanami A Nagashima T Ono M Miyoshi H Okano HJ Ito M Noninvasive and real-time assessment of reconstructed functional human endometrium in NOD/SCID/gamma c(null) immunodeficient mice Proc Natl Acad Sci USA 2007 104 1925 1930 10.1073/pnas.0604310104 17261813 PMC1794295

[B26] Grummer R Animal models in endometriosis research Hum Reprod Update 2006 12 641 649 10.1093/humupd/dml026 16775193

[B27] Brar AK Kanda Y Kessler CA Cedars MI Handwerger S N5 endometrial stromal cell line: a model system to study decidual prolactin gene expression In Vitro Cell Dev Biol Anim 1999 35 150 154 10.1007/s11626-999-0017-5 10476911

[B28] Chapdelaine P Kang J Boucher-Kovalik S Caron N Tremblay JP Fortier MA Decidualization and maintenance of a functional prostaglandin system in human endometrial cell lines following transformation with SV40 large T antigen Mol Hum Reprod 2006 12 309 319 10.1093/molehr/gal034 16556676

[B29] Barbier CS Becker KA Troester MA Kaufman DG Expression of exogenous human telomerase in cultures of endometrial stromal cells does not alter their hormone responsiveness Biol Reprod 2005 73 106 114 10.1095/biolreprod.104.035063 15772261

[B30] Samalecos A Reimann K Wittmann S Schulte HM Brosens JJ Bamberger AM Gellersen B Characterization of a novel telomerase-immortalized human endometrial stromal cell line, St-T1b Reprod Biol Endocrinol 2009 7 76 10.1186/1477-7827-7-76 19619280 PMC2719639

[B31] Cloke B Huhtinen K Fusi L Kajihara T Yliheikkila M Ho KK Teklenburg G Lavery S Jones MC Trew G The androgen and progesterone receptors regulate distinct gene networks and cellular functions in decidualizing endometrium Endocrinology 2008 149 4462 4474 10.1210/en.2008-0356 18511503 PMC5393297

[B32] Samalecos A Gellersen B Systematic expression analysis and antibody screening do not support the existence of naturally occurring progesterone receptor (PR)-C, PR-M, or other truncated PR isoforms Endocrinology 2008 149 5872 5887 10.1210/en.2008-0602 18617611

[B33] Gellersen B Fernandes MS Brosens JJ Non-genomic progesterone actions in female reproduction Hum Reprod Update 2009 15 119 138 18936037 10.1093/humupd/dmn044

[B34] Richards RG Brar AK Frank GR Hartman SM Jikihara H Fibroblast cells from term human decidua closely resemble endometrial stromal cells: induction of prolactin and insulin-like growth factor binding protein-1 expression Biol Reprod 1995 52 609 615 10.1095/biolreprod52.3.609 7756454

[B35] Dimitrov R Kyurkchiev D Timeva T Yunakova M Stamenova M Shterev A Kyurkchiev S First-trimester human decidua contains a population of mesenchymal stem cells Fertil Steril 2010 93 210 219 10.1016/j.fertnstert.2008.09.061 19006798

[B36] Gao JG Mazella J Tseng L Activation of the human IGFBP-1 gene promoter by progestin and relaxin in primary culture of human endometrial stromal cells Mol Cell Endocrinol 1994 104 39 46 10.1016/0303-7207(94)90049-3 7529731

[B37] Tseng L Gao JG Chen R Zhu HH Mazella J Powell DR Effect of progestin, antiprogestin, and relaxin on the accumulation of prolactin and insulin-like growth factor-binding protein-1 messenger ribonucleic acid in human endometrial stromal cells Biol Reprod 1992 47 441 450 10.1095/biolreprod47.3.441 1380842

[B38] Boe R Gjertsen BT Doskeland SO Vintermyr OK 8-Chloro-cAMP induces apoptotic cell death in a human mammary carcinoma cell (MCF-7) line Br J Cancer 1995 72 1151 1159 10.1038/bjc.1995.479 7577461 PMC2033955

[B39] Dowd DR Miesfeld RL Evidence that glucocorticoid- and cyclic AMP-induced apoptotic pathways in lymphocytes share distal events Mol Cell Biol 1992 12 3600 3608 1378529 10.1128/mcb.12.8.3600-3608.1992 PMC364626

[B40] Mote PA Balleine RL McGowan EM Clarke CL Colocalization of progesterone receptors A and B by dual immunofluorescent histochemistry in human endometrium during the menstrual cycle J Clin Endocrinol Metab 1999 84 2963 2971 10.1210/jc.84.8.2963 10443705

[B41] Mulac-Jericevic B Conneely OM Reproductive tissue selective actions of progesterone receptors Reproduction 2004 128 139 146 10.1530/rep.1.00189 15280552

[B42] Labied S Kajihara T Madureira PA Fusi L Jones MC Higham JM Varshochi R Francis JM Zoumpoulidou G Essafi A Progestins regulate the expression and activity of the forkhead transcription factor FOXO1 in differentiating human endometrium Mol Endocrinol 2006 20 35 44 16123151 10.1210/me.2005-0275

